# Identification of Colitis and Cancer in Colon Biopsies by Fourier Transform Infrared Spectroscopy and Chemometrics

**DOI:** 10.1100/2012/936149

**Published:** 2012-05-03

**Authors:** Xiang Li, Qing-Bo Li, Guang-Jun Zhang, Yi-Zhuang Xu, Xue-Jun Sun, Jing-Sen Shi, Yuan-Fu Zhang, Jin-Guang Wu

**Affiliations:** ^1^College of Instrument Science and Opto-Electronics Engineering, Beihang University, Beijing 100083, China; ^2^College of Chemistry and Molecular Engineering, Peking University, Beijing 100871, China; ^3^Department of General Surgery, First Hospital of Xi'an Jiaotong University, Shanxi, Xi'an 710061, China

## Abstract

Cancer is a disease that does great harms to the health of human beings. FT-IR spectroscopy could identify variability at the molecular level in biological specimens. It is a rapid and noninvasive method, which could be used intraoperatively to modify surgical procedures. The aim of this paper is to identify and separate cancer from colitis in endoscopic colon biopsies through the use of FT-IR spectroscopy. A total of 88 endoscopic colon samples, including 41 cases of colitis and 47 cases of colon cancer, were obtained. Specimens were placed on an ATR accessory linked to FT-IR spectrometer with a MCT detector for greater stability and sensitivity. Later, specimens were sent for the histological examination as the reference in the spectral analysis. 41 colitis and 47 cancer specimens were compared. Spectra preprocessed with smoothing and normalization were used for discrimination analysis. PCA was processed to simplify the spectrum data set. Naive Bayes classifier model was constructed for diagnostic classification. Leave-one-out cross-validation method was utilized to assess the discrimination results. The sensitivity of FT-IR detection for cancer achieves 97.6%. The results showed that colon cancer could be distinguished from colitis with high accuracy using FT-IR spectroscopy and chemometrics.

## 1. Introduction

Cancer is a disease that does great harms to the health of human beings. The survival of patients depends largely on the detection of cancer at an early stage. It is of great importance to explore the early cancer diagnosis method. But when the changes in morphology can be seen under light microscope, there have been millions of cancer cells. In the process of carcinogenesis, nuclear acids, proteins, carbohydrates, and other biomolecules generate significant changes in their molecular structures. Fourier transform infrared (FT-IR) spectroscopy is a powerful tool to detect the changes of molecular structure and composition [1–3]. Therefore, it is possible for the FT-IR spectral analysis technology to become a rapid, noninvasive, and convenient method to detect tumors at the precarcinogenesis stage [4, 5]. At present, with the development of biospectroscopy and spectral analysis technology, the application of FT-IR spectroscopy in distinguishing malignant tissues from normal ones has become a focus [6–10]. Also, great progresses have been made in the research of cancer detection using FT-IR spectroscopy [11–17]. 

FT-IR spectroscopy can effectively provide chemical variation information about the structure and the composition of biological materials at molecular level. FT-IR technology makes it possible to detect inflammatory and cancer of the enteroscopic biopsies. It indicated that FT-IR method has the opportunity to develop as a new technique for enteroscope examination. We believe that noninvasive, rapid, accurate, and convenient analysis of colon tissues can be performed with Fourier-transform midinfrared spectroscopy if the mid-infrared fiber optics and colon endoscopy technologies can be combined successfully. The fundamental study on the application of chemometrics to the identification of colon biopsies, obtained from enteroscopy detection and measured in vitro using FT-IR spectrometer, was performed in this paper.

## 2. Theory

### 2.1. Principal Component Analysis

One of the difficulties in spectral analysis is that spectral data usually has too many variables. Fortunately, in spectrum data sets, groups of variables often move together. The absorption bands in neighborhood are related to each other [18]. Thus, here is plenty of redundancy of information in spectrum data set.

Principal component analysis (PCA) is a quantitatively mathematical procedure for achieving simplification. The method generates a new set of variables, called principal components. Each principal component is a linear combination of the original variables. All the principal components are orthogonal to each other, so there is no redundant information. The principal components as a whole form an orthogonal basis for the space of the data. This linear transformation has been widely used in data analysis and compression.

The first principal component is a single axis in space. When each observation is projected on that axis, the resulting values form a new variable. And the variance of this variable is the maximum among all possible choices of the first axis.

The second principal component is another axis in space, perpendicular to the first. Projecting the observations on this axis generates another new variable. The variance of this variable is the maximum among all possible choices of this second axis. And the rest principal components are also settled as above [19].

The full set of principal components is as large as the original set of variables. But it is commonplace for the sum of the variances of the first few principal components to explain most information of the original data.

### 2.2. Naïve Bayes classifier

A Naïve Bayes Classifier (NBC) is a simple probabilistic classifier based on applying Bayes' theorem with strong independence assumptions [20]. In simple terms, a Naïve Bayes classifier assumes that the presence of a particular feature of a class is unrelated to the presence of any other feature, given the class variable. It classifies data in two steps.

First, using the training samples, the method estimates the parameters of a probability distribution, assuming that features are conditionally independent given the class. Naïve Bayes classifiers can be trained very efficiently in a supervised learning setting. In many practical applications, parameter estimation for Naïve Bayes models uses the method of maximum likelihood [21, 22].

Then, for any unseen test sample, the method computes the posterior probability of that sample belonging to each class. The method then classifies the test sample according to the largest posterior probability.

An advantage of the Naïve Bayes classifier is that it only requires a small amount of training data to estimate the parameters (means and variances of the variables) necessary for classification. Because independent variables are assumed, only the variances of the variables for each class need to be determined and not the entire covariance matrix.

## 3. Experimental

### 3.1. Patients

Informed consent was obtained from each patient prior to the study. A total of 89 colon biopsies were obtained from clinical detection in the Medical Division of the First Hospital of Xi'an Jiaotong University, China. There were 43 women and 45 men, aged between 21 and 76 years (mean 53.7 years old). One endoscopic pinch biopsy of 1–3 mm in diameter was obtained from each patient. According to the results of pathological detection, the studied samples consisted of 41 cases of colitis and 47 cases of cancer.

### 3.2. Instrument

An attenuated total reflectance (ATR) accessory linked to a WQD-500 FT-IR spectrometer (Beijing No. 2 Optical Instrument Factory, Beijing, China) was used. The ATR accessory was made of ZnSe crystal. The FT-IR spectrometer is equipped with a liquid nitrogen-cooled mercury cadmium telluride detector.

### 3.3. Spectral Measurements

Specimens were frozen and transported to the laboratory. The sample was thawed at room temperature for about 3–5 minutes, and placed on the ATR accessory to record the spectrum using WQD-500 FT-IR spectrometer. The background spectrum was collected at first. 32-coadded scans at a resolution of 4 cm^−1^, with a normal range from 1000 to 4000 cm^−1^, were performed. It took about 1-2 minutes to measure the spectrum noninvasively. After the spectra of the samples were recorded, the samples were stored in liquid nitrogen and sent for the histological examination as the reference in the spectral analysis.

### 3.4. Computer Programs

The Matlab programs used for smoothing, normalization, PCA, and NBC discrimination analysis were developed in our laboratory. These programs can be compiled on any digital computer and are available from the corresponding author.

## 4. Results and Discussion

In the process of measuring the spectra, the spectra contain not only the useful information of molecular structure and component of the measured samples, but also the noises such as the high-frequency random noise, baseline drift, light scattering, and so forth. These additional noises must be eliminated; otherwise, they will affect the result of the discrimination.

**Figure 1:**
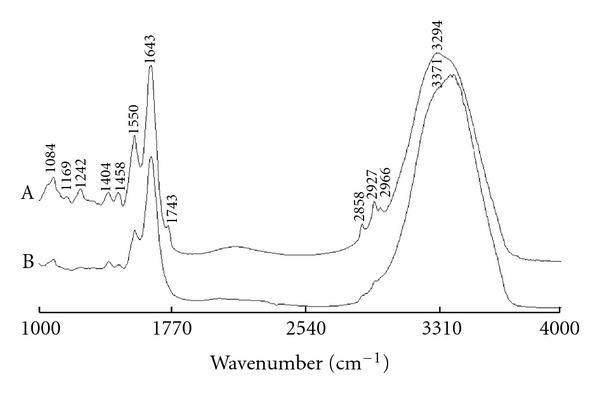
Mean FT-IR spectra of colon biopsies. *Trace (A)*, colitis; *Trace (B)*, colon cancer.

**Figure 2:**
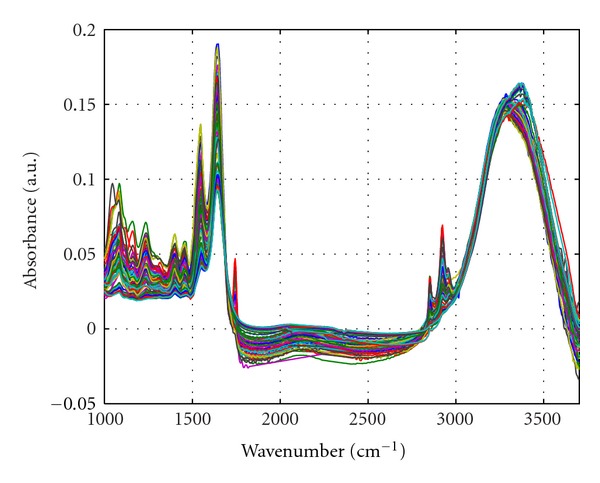
Preprocessed spectra with smoothing and normalization (1000–1766 cm^−1^, 2731–3695 cm^−1^).

Firstly, smoothing is utilized to filter high-frequency noise.  Figure 1 showed the mean spectra of colitis and colon cancer biopsies (1000–4000 cm^−1^). The absorption band of CO2 and the spectral range of 3696–4000 cm^−1^ were excluded, which contained little useful information for measurement. The spectral data of these two ranges were mean normalized, respectively. Preprocessed spectra with smoothing and normalization are shown in Figure 2. And, the PCA was used to simplify the spectrum data set. Only the first 5 principal components were reserve, which expressed over 90% information of original data set. And the rest data was eliminated. Apparently, the PCA could reduce a lot of computation of NBC. Then the NBC model was built for classification of cancer and colitis samples. Cross-validation was utilized to evaluate the discrimination results. The FT-IR analysis results (Table 1) were as follows: among the 47 cases of colitis samples, 33 cases were correctly distinguished while 14 samples were misjudged; among the 41 cases of cancer samples, 40 cases were well judged while only 1 case was misjudged. Total correctness was 82.8%. Statistical results of colon biopsies using FT-IR spectroscopy (Table 2) showed that sensitivity of cancer diagnosis was 97.6%, specificity of cancer diagnosis for 70.2%, predictive value of a positive test of cancer diagnosis for 74.1%, and predictive value of a negative test of cancer diagnosis for 97.1%.

There are different spectral characteristics between colitis and malignant colon enteroscope samples in the FT-IR spectra. These spectral features are related to the changes of structure and composition of biological molecular in tissue cell. The mean spectra of colitis and cancer got from enteroscope detection are illustrated in Figure 1. The spectral features of these two types of colon biopsies were as follows: C=O band near 1743 cm^−1  ^was assigned to the fat in tissues, and C-H-stretching vibration bands near 2966 cm^−1^, 2927 cm^−1^, and 2858 cm^−1  ^were related to lipid and fat content, and these bands usually decreased and even disappeared in the spectra of malignant tissues, for that the fat in the malignant tissue is consumed because of the necessary increased nutritional and energy requirement in the development of the carcinoma. ~1643 cm^−1^ absorption peak belonged to amide I band of protein and H–O–H deformation vibration of water. ~1550 cm^−1^ absorption peak was assigned to amide II band of protein. The relative intensity of amide II band to ~1643 cm^−1^ absorption peak decreased in the spectra of malignant colon tissues than those in colitis biopsies. The intensity of ~1460 cm^−1^ peak was weaker than that of ~1400 cm^−1^ peak in the spectra of the cancerous samples. The peak at ~1460 cm^−1^ was stronger than or equal to that of ~1400 cm^−1^ in the spectra of colitis samples.

## 5. Conclusion

In conclusion, the results indicate that colon cancer can be distinguished from colitis with high accuracy using FT-IR spectroscopy and chemometrics. This should be extended to analysis of cancer versus normal colon or polyps. This technique has promise for in vivo diagnosis with the development of endoscopic FT-IR miniprobes and is practical for immediate diagnosis at endoscopy.

## Figures and Tables

**Table 1:** Comparison of FT-IR results with histological examination.

Histologic examination	FT-IR results	
	Cancer	Colitis
Cancer	40	1
Colitis	14	33

**Table 2:** Results of statistical analysis of detection of colon biopsies by FT-IR spectroscopy.

	Sensitivity, %	Specificity, %	Predictive value of a positive test, %	Predictive value of a negative test, %
Cancer	97.6	70.2	74.1	97.1
